# Characterising smokers of menthol and flavoured cigarettes, their attitudes towards tobacco regulation, and the anticipated impact of the Tobacco Products Directive on their smoking and quitting behaviours: The EUREST-PLUS ITC Europe Surveys

**DOI:** 10.18332/tid/96294

**Published:** 2018-12-14

**Authors:** Mateusz Zatoński, Aleksandra Herbeć, Witold Zatoński, Krzysztof Przewoźniak, Kinga Janik-Koncewicz, Ute Mons, Geoffrey T. Fong, Tibor Demjén, Yannis Tountas, Antigona C. Trofor, Esteve Fernández, Ann McNeil, Marc Willemsen, Karin Hummel, Anne C. K. Quah, Christina N. Kyriakos, Constantine I. Vardavas, on behalf of the EUREST-PLUS consortium

**Affiliations:** 1 Health Promotion Foundation (HPF), Warsaw, Poland; 2 Department of Health Services Research and Policy, London School of Hygiene and Tropical Medicine, London, United Kingdom; 3 European Observatory of Health Inequalities, President Stanisław Wojciechowski State University of Applied Sciences, Kalisz, Poland; 4 UK Centre for Tobacco and Alcohol Studies, London, United Kingdom; 5 Department of Behavioural Science and Health, University College London, London, United Kingdom; 6 Maria Skłodowska-Curie Institute - Oncology Center, Warsaw, Poland; 7 Cancer Prevention Unit and WHO Collaborating Centre for Tobacco Control, German Cancer Research Center (DKFZ), Heidelberg, Germany; 8 Department of Psychology & School of Public Health and Health Systems, University of Waterloo (UW), Waterloo, Canada; 9 Ontario Institute for Cancer Research, Toronto, Canada; 10 Smoking or Health Hungarian Foundation (SHHF), Budapest, Hungary; 11 University of Athens (UoA), Athens, Greece; 12 University of Medicine and Pharmacy ‘Grigore T. Popa’ Iasi, Iasi, Romania; 13 Aer Pur Romania, Bucharest, Romania; 14 Tobacco Control Unit, Catalan Institute of Oncology (ICO), and Cancer Control and Prevention Group, Bellvitge Biomedical Research Institute (IDIBELL), L’Hospitalet, Catalonia, Spain; 15 School of Medicine and Health Sciences, University of Barcelona, Catalonia, Spain; 16 National Addiction Centre, King’s College London, London, United Kingdom; 17 Department of Health Promotion, Maastricht University, Maastricht, the Netherlands; 18 Department of Psychology, University of Waterloo (UW), Waterloo, Canada; 19 European Network for Smoking and Tobacco Prevention (ENSP), Brussels, Belgium; 20 University of Crete (UoC), Heraklion, Greece

**Keywords:** menthol cigarettes, flavoured cigarettes, ban on additives, cross-sectional study

## Abstract

**INTRODUCTION:**

Little research exists on the sociodemographic characteristics of menthol and flavoured cigarette (MFC) smokers in Europe. This study assessed the proportion of MFC smokers in Europe, their sociodemographic characteristics, and their attitudes towards tobacco control measures.

**METHODS:**

Cross-sectional data were collected in 2016 among 10760 adult current smokers from 8 European countries (ITC Europe Project and EUREST-PLUS). Smokers of menthol, other flavoured, unflavoured tobacco, or no usual brand were compared on sociodemographic characteristics, attitudes towards a range of tobacco control measures (e.g. ban on flavouring), and on intentions regarding their smoking behaviour following the ban on flavoured tobacco. Data were analysed in SPSS Complex Samples Package using univariate analyses.

**RESULTS:**

Among the respondents, 7.4% smoked menthol cigarettes and 2.9% other flavoured tobacco, but large differences existed between countries (e.g. 0.4% smokers smoked menthol cigarettes in Spain vs 12.4% in England). Compared to other groups, menthol cigarette smokers were younger, more likely to be female, better educated, had higher household income, and smoked fewer cigarettes (all p<0.001). A quarter of menthol smokers supported a ban on additives, compared with almost half of all other smokers (p<0.001). In case of a ban on flavourings, around a fifth of all MFC smokers intended to switch to another brand, and a third to reduce the amount they smoked or to quit smoking, but there was no consistent pattern across MFC smokers among the countries.

**CONCLUSIONS:**

The ban on flavourings introduced by the EU Tobacco Products Directive (extended to 2020 for menthols) will affect one in ten smokers in the countries surveyed, which provides an opportunity for targeting these groups with cessation programmes. However, smokers of menthol and flavoured cigarettes in the different European countries are a heterogeneous group and may need different approaches.

## INTRODUCTION

The development of flavoured cigarettes is a relatively recent innovation in a long line of product innovations carried out by transnational tobacco companies (TTCs), created in the hope of increasing their market share in an increasingly regulated environment^1^. The most popular variety of flavoured cigarettes are menthol cigarettes, first patented in the United States in 1925, but their mass distribution and marketing only began^2^ in earnest in the 1960s. Today, cigarettes are produced with a variety of flavouring agents, including sweet flavours, such as strawberry, honey, or apple^3^.

Research based on tobacco industry internal documents, along with trends data, suggest that flavoured brands have been marketed using messages directly targeting youth^4,5^. In addition, several studies found that flavoured cigarettes, due to the masking of the harsh taste of tobacco, might be perceived as less harmful by smokers^4,6^. It has also been suggested that the cooling effect of menthol cigarettes may reduce smokers’ perception of irritation from tobacco smoke, increasing overall nicotine and smoke intake^7^. Finally, flavoured tobacco smokers have been found to be most at risk of developing sustained tobacco-use behaviours throughout their lifetime^4,8^. Despite opposition from the TTCs, several countries have made progress in limiting the exploitation of tobacco ingredients. In 2012, Brazil became the first country to ban menthol and almost all other additives in tobacco products^9^.

Very little research exists on both the sociodemographic characteristics of menthol and flavoured cigarette (MFC) smokers, and on their attitudes towards tobacco control regulation, in particular towards restrictions on the sales of menthol and flavoured cigarettes. The vast majority of existing studies are based on US data^7,10-16^. While some recent tobacco industry estimates conducted for commercial purposes exist for Europe^17,^ academic and scientific research is sparse, and it is hard to assess the accuracy of industry statistics. One notable exception is the work of Giovino et al.^18^, which reported menthol cigarette share for 19 European countries. However, data used for this study had been collected as far back as 1999–2001; they showed a menthol market share ranging from 1–2% in Germany, Hungary and Italy, to 3–5% in the UK, Norway and Czech Republic, over 10% in Poland (11.7%), Romania (15.4%) and Finland (18.2%)^18^.

The scarcity of research in this area is particularly worrying in the context of the decision of the European Union (EU) to introduce the new Tobacco Products Directive (TPD) that bans products with flavours as of May 2016 (while allowing the retailers to sell their stocks until May 2017) with a transitional period for menthol cigarettes until 2020^19^. The ban on MFCs could be an opportunity to wean smokers of MFCs off tobacco altogether, as they will be adapting their smoking behaviour in the aftermath of the ban. However, in order to design and implement effective interventions, a better understanding of who are the MFC smokers is needed. In addition, the level of support for a new tobacco control policy is important to measure, as it is a predictor for the rates of compliance once the regulation is implemented^20^. The lack of data leaves the TTCs free reign to make claims that regulation of ingredients will not help to lower smoking rates, but rather constitute a driver of illicit cigarette trade^21^. A 2016 WHO report on menthol regulation called for more surveys on the attitudes of flavoured cigarette smokers towards regulation to be conducted in regions other than the US^19^.

In order to address this gap in research, the current study aims to identify the prevalence of MFC use and sociodemographic characteristics of MFC smokers in Europe, as well as to examine attitudes towards tobacco control measures, and intentions to comply with the TPD bans on cigarettes with characterising flavours (introduced in May 2016) and on menthol cigarettes (to be introduced in 2020).

## METHODS

### Design

The current study is part of the European Regulatory Science on Tobacco: Policy Implementation to Reduce Lung Disease Project (EUREST-PLUS; registered on Clinicaltrials.gov: NCT02773836), the goal of which is to evaluate the implementation of the EU TPD (2014/40/EU)^22^. This study uses cross-sectional data from adult (age 18 years or older) current smokers from Wave 1 (2016) of the International Tobacco Control Policy Evaluation Project (ITC) 6 European Country (6E) Survey involving Germany, Greece, Hungary, Poland, Romania, and Spain, as well as Wave 10 (2016) from the ITC Netherlands (NL10) Survey, and data from England collected as part of Wave 1 (2016) of the Four Country Smoking and Vaping (4CV1) Survey^23^. The ITC Project is a prospective cohort study that evaluates the psychosocial and behavioural effects of tobacco control policies, both at the State and international level across 29 countries. All ITC surveys, including EUREST-PLUS surveys, are developed using the same approach and policy framework, and they include questions that are worded in the same manner, or are functionally equivalent, to enable comparisons across countries. Furthermore, the questions are translated by teams of professionals and checked for accuracy by the regional project leaders^24,25^. Nearly all ITC countries have longitudinal data available, including those of the ITC 6E Survey. For more information about ITC waves, see the ITC website^26^. This paper reports on cross-sectional data collected in 2016 from European countries in order to characterise smokers at the initiation of TPD, which includes data from ITC 6E Survey.

The ITC 6E Survey involved face-to-face computer-assisted personal interviews (CAPI) conducted between June and September 2016 (after the introduction of the ban on characterising flavours in May 2016, except for menthol). Both the Netherlands and England data were collected through internet surveys. Fieldwork for the Netherlands took place between November and December 2016, while the England fieldwork was conducted between July and November 2016. All these countries combined will be referred to as 8E in the manuscript. The study was approved by the Research Ethics Board of the University of Waterloo, Ontario, Canada and by local ethics boards in the participating countries. Participants had to provide informed consent before participating.

### Study population

The 8E sample included in the study comprised 10760 adult cigarette smokers (age 18 years or older, reporting that they had smoked at least 100 factory-made or roll-your-own cigarettes in their lifetime, and were currently smoking at least monthly, regardless of cigarette type).

Each 6E nationally representative country sample comprised approximately 1000 participants (ranging from 1000 in Greece and Hungary, to 1006 in Poland). Sampling followed the Eurobarometer Survey sampling design using geographic strata determined by Nomenclature of Territorial Units for Statistics (NUTS) regions crossed with degree of urbanization (urban, intermediate, rural). Approximately 100 area clusters were sampled in each country, with the aim of recruiting 10 adult smokers per cluster. Clusters were allocated to strata proportionally to age 18 years and older population size. Within each cluster, household addresses were sampled using a random walk design. One randomly selected male smoker and one randomly selected female smoker were chosen for interview from a sampled household, where possible. Screening of households continued until the required number of smokers from the cluster had been interviewed. Further details on survey methodology and response rates of ITC 6E are available elsewhere^22,24^.

The nationally representative sample in England, had a retention rate from Wave 10 of ITC Four Country (4C10) Survey in the UK of 35.7% and a response rate of new recruits of 15.2%, and comprised: 1) re-contact smokers and quitters living in England who participated in the 4C10 Survey, regardless of e-cigarette use; 2) newly recruited current smokers and recent quitters (quit smoking in the past 24 months) from a commercial online panel, regardless of e-cigarette use; and 3) newly recruited current e-cigarette users (use at least weekly) from a commercial online panel. In sampling, quotas obtained from national survey data for region crossed with male/female were applied to 2) and 3). Only current tobacco smokers were included in the present analysis (n=3536). Further details on survey methodology are available elsewhere^23^.

The surveyed sample in the Netherlands included 1696 respondents age 15 years or older recruited as cigarette smokers from Wave 10 of the ITC Netherlands Project, who were part of a probability-based web database^27^ . The nationally representative sample included 1318 respondents who had also participated in Wave 9, and 378 new respondents recruited to replenish dropouts (response rate for recontact was 67% and replenishment was 45%)^28^. Only current adult (age 18 years or older) smokers were included in the analysis (n=1213). Further details on survey methodology are available elsewhere^29^.

### Measurements

#### Cigarette flavour

The type of cigarettes smoked was assessed through two questions in the survey. First, participants were asked: ‘do you have a usual brand of cigarettes’; with response ‘yes/no’. Those who had a usual brand were then asked: ‘which is your usual flavour of cigarettes smoked?’; with choices being tobacco and menthol, tobacco and other flavour, just tobacco, unknown, or refused to answer. Participants who reported not having a usual brand, those who provided no answer to the question on usual brand, and those who did not select any of the flavour answers were all classified as ‘no usual brand’. In effect, participants were categorised into four groups: menthol, other flavoured, unflavoured tobacco, no usual brand.

#### Dependence level

Dependence was assessed as cigarettes smoked per day.

#### Sociodemographics

Data were collected on the country (Germany, Greece, Hungary, Netherlands, Poland, Romania, Spain, England), sex (male, female), age (categorized into age groups: 18–24, 25–39, 40–54, 55+ years old), educational level (derived variable: low, medium, high), and household income level (derived variable: low, medium, high, not reported).

#### Attitudes to tobacco control measures

We assessed attitudes towards the following tobacco control measures: ban on additives (‘Would you support or oppose a law that banned all additives, including flavourings, from cigarettes?’), smoking ban in restaurants (‘Do you support or oppose a complete smoking ban inside restaurants?’), smoking bans in bars and pubs (‘Do you support or oppose a complete smoking ban inside drinking establishments such as pubs and bars?’), more regulation on tobacco (‘Tobacco products should be subject to more rules and regulations’), and plain packaging (‘Tobacco companies should be required to sell cigarettes in plain packages, i.e. in packs without the usual brand colours and symbols, but keeping the warning labels’). The answer options were: strongly support, support, oppose, strongly oppose, refused, or don’t know. Answers ‘strongly support’ and ‘support’ were classified as ‘support’ for the measure, while all others were classified as lack of support.

#### Intentions regarding smoking behaviour following bans on flavourings

Smokers of menthol cigarettes were asked: ‘Which of the following describes what you would be most likely to do if the use of menthol in cigarettes was banned by the government?’; while other flavoured cigarette smokers were asked: ‘Which of the following describes what you would be most likely to do if flavourings in cigarettes, such as menthol, chocolate or vanilla, were banned by the government?’; with answer options: switch to another (non-menthol, non-flavoured) brand, find a way to get menthol (or flavoured) cigarettes, reduce the amount you smoke, quit smoking entirely, do something else, refused or don’t know.

### Analysis

For the main analyses data from all countries were pooled and analysed together using Complex Samples package in SPSS 23.00 that accounted for the sampling procedure in each country, as well as weighted the data for sex and age to make the data more representative for each country’s population of smokers^23,24,29^. Missing data on cigarettes smoked per day (1.1%) and education (1.2%) were not imputed and were excluded on a case-by-case basis. Income data were missing from 18.8% of participants, and this group is included as a separate group (‘income not reported’). For dichotomous variables on support for tobacco control, answer options ‘don’t know’ and ‘refused’ were rare (<4.6% combined, except for 18.3% for ban on all additives) and were classified as ‘no support’. This conservative approach was chosen in order not to overestimate the popularity of such measures. Smokers of different cigarette flavours were compared on outcomes of interest using Complex Samples Crosstabs (overall tests of association using Rao-Scott chi-squared) option in SPSS, and the calculated 95% Confidence Intervals (CIs) for these analyses are presented. All tests were two-sided and a p-value of 0.05 was considered to be statistically significant.

## RESULTS

### Prevalence of use of different flavours of cigarettes

Table 1 presents the weighted prevalence of different flavours of cigarettes across the 8E countries, and for men and women separately in each country. Across the surveyed countries a minority (7.2%) of smokers indicated menthol as their usual brand. Considerable difference existed between sexes, with 4.4% of men and 11.1% of women smoking menthol cigarettes. The countries with the highest menthol use were England (12.4%), Poland (10.2%), and Romania (7.8%). The proportion was lowest in Spain (0.4%). In all the surveyed countries menthol use was higher among women, with rates among females as high as 17.5% in England and 17.3% in Poland.

**Table 1:** Prevalence of flavour of usual brand of cigarettes in the 8E countries, for men and women together and separately

	*Menthol*	*Other flavoured*	*Unflavoured tobacco*	*No usual brand^a^*

	*% (95% CI)*			
**Germany**	**2.1 (1.4–3.2)**	**4.5 (2.6–7.7)**	**74.6 (68.0–80.2)**	**18.8 (14.1–24.5)**
Men	1.4 (0.7–2.7)	4.1 (2.4–7.0)	75.8 (69.0–81.4)	18.7 (13.7–25.0)
Women	3.3 (2.0–5.5)	5.2 (2.6–10.2)	72.7 (64.8–79.4)	18.8 (13.6–25.4)
**Greece**	**2.6 (1.4–4.9)**	**5.8 (3.4–9.8)**	**87.3 (82.8–90.7)**	**4.3 (2.7–6.6)**
Men	2.5 (1.3–5.0)	6.7 (3.6–12.1)	86.3 (80.7–90.5)	4.5 (2.9–6.8)
Women	2.8 (1.3–5.6)	4.9 (2.7–8.6)	88.3 (83.3–92.0)	4.0 (2.2–7.4)
**Hungary**	**4.9 (3.6–6.8)**	**2.2 (1.4–3.3)**	**83.9 (79.1–87.9)**	**9.0 (5.7–13.8)**
Men	2.6 (1.5–4.4)	1.5 (0.7–3.0)	85.4 (79.6–89.7)	10.5 (6.7–16.3)
Women	8.3 (5.7–11.9)	3.2 (1.8–5.5)	81.9 (76.4–86.3)	6.6 (3.9–11.1)
**Poland**	**10.2 (8.2–12.7)**	**3.0 (1.8–4.8)**	**59.3 (53.9–64.6)**	**27.5 (22.3–33.3)**
Men	4.5 (2.8–7.1)	2.3 (1.1–4.7)	62.6 (55.6–69.0)	30.6 (24.3–37.8)
Women	17.3 (13.9–21.5)	3.8 (2.2–6.6)	55.3 (49.5–61.0)	23.5 (18.4–29.4)
**Romania**	**7.8 (6.0–10.1)**	**1.5 (0.9–2.7)**	**76.1 (71.7–80.1)**	**14.5 (11.1–18.7)**
Men	5.4 (3.4–8.5)	1.5 (0.7–3.0)	75.6 (70.0–80.3)	17.5 (13.0–23.1)
Women	11.2 (7.9–15.8)	1.6 (0.7–3.3)	77.0 (70.8–82.2)	10.2 (7.1–14.6)
**Spain**	**0.4 (0.2–1.1)**	**5.9 (2.4–13.8)**	**84.9 (78.0–90.0)**	**8.7 (6.0–12.4)**
Men	0.1 (0.0–0.9)	6.3 (2.6–14.3)	82.9 (75.1–88.7)	10.6 (6.9–16.1)
Women	0.8 (0.3–2.4)	5.5 (2.1–13.5)	87.6 (80.6–92.3)	6.1 (3.9–9.3)
**England**	**12.4 (11.0–13.9)**	**2.2 (1.6–3.0)**	**75.1 (73.2–76.9)**	**10.3 (9.1–11.7)**
Men	8.0 (6.6–9.8)	3.1 (2.1–4.4)	76.8 (74.1–79.3)	12.1 (10.2–14.2)
Women	17.5 (15.1–20.0)	1.2 (0.7–2.1)	73.1 (70.3–75.7)	8.2 (6.8–9.9)
**NL**	**6.5 (5.1–8.3)**	**0.3 (0.1–0.9)**	**82.3 (79.7–84.7)**	**10.9 (9.0–13.1)**
Men	2.5 (1.3–4.6)	0.1 (0.0–1.0)	86.8 (83.3–89.7)	10.6 (8.0–13.8)
Women	10.5 (8.1–13.4)	0.4 (0.1–1.7)	78.0 (74.0–81.5)	11.2 (8.5–14.5)
**Total**	**7.4 (6.8–8.1)**	**2.9 (2.3–3.7)**	**77.4 (76.0–78.7)**	**12.3 (11.3–13.4)**
Men	4.4 (3.8–5.1)	3.1 (2.4–4.0)	78.5 (76.8–80.1)	13.9 (12.6–15.4)
Women	11.1 (10.1–12.3)	2.6 (2.0–3.4)	75.9 (74.2–77.5)	10.3 (9.3–11.5)

a In the unweighted sample this group included respondents who indicated that they have no usual brand (n=1277), who refused to provide an answer or responded ‘don’t know’ to the question on whether they have a usual brand (n=48), and who refused or responded ‘don’t know’ to the question on the flavour of their usual brand (n=45). CI: confidence interval.

Other flavoured cigarettes were less popular than menthol cigarettes. The difference between sexes was small, with 3.1% of male and 2.6% of female respondents indicating other flavoured cigarettes as their usual brand. The countries with the highest use of other flavoured cigarettes were Spain (5.9%) and Greece (5.8%), while the proportion was lowest in the Netherlands (0.3% of smokers).

Overall, the combined prevalence of MFC smokers among smokers was between 5% and 15% in all the countries surveyed, with the average over 10%. Among women this was 13.7% and among men 7.5%. The combined prevalence was highest in England (14.6%), followed by Poland (13.2%).

### Sociodemographic characteristics

There were statistically significant differences on all these measures based on sociodemographic characteristics as well as dependence levels between smokers of the different flavours (Tables 2, 3 and 4, all overall chi-squared tests had p<0.001). Across the surveyed countries, menthol cigarette smokers tended to be younger, more likely to be female, better educated, living with a higher household income, and smoking fewer cigarettes. The educational and dependence (cigarettes smoked per day) profiles of smokers of other flavoured cigarettes were more similar to those of unflavoured tobacco smokers than those of menthol cigarette smokers. Smokers of other flavoured cigarettes tended to have lower household incomes then menthol and unflavoured cigarette smokers.

**Table 2:** Prevalence of different cigarette flavours across sociodemographic characteristics and dependence levels

	*Menthol (n=795.2)^a^*	*Other flavoured (n=311.5)^a^*	*Unflavoured tobacco (n=8298.3)^a^*	*No usual brand (n=1321.5)^a^*	*p^c^*

	*% (95% CI)*				
**Sex**					
Male	4.4 (3.8–5.1)	3.1 (2.4–4.0)	78.5 (76.8–80.1)	13.9 (12.6–15.4)	<0.001
Female	11.1 (10.1–12.3)	2.6 (2.0–3.4)	75.9 (74.2–77.5)	10.3 (9.3–11.5)	
**Age (years)**					
18–24	11.6 (9.7–13.9)	3.4 (2.1–5.7)	67.6 (64.3–70.8)	17.3 (15.0–19.9)	<0.001
25–39	9.6 (8.3–11.0)	2.7 (2.0–3.6)	76.3 (74.0–78.4)	11.5 (9.9–13.3)	
40–54	5.5 (4.7–6.5)	3.4 (2.4–4.8)	80.4 (78.3–82.3)	10.7 (9.3–12.3)	
55+	5.1 (4.2–6.1)	2.4 (1.8–3.1	79.7 (77.7–81.6)	12.9 (11.2–14.7)	
**Level of education**					
Low	3.4 (2.9–4.1)	3.3 (1.9–5.7)	81.1 (78.6–83.4)	12.1 (10.6–13.9)	<0.001
Moderate	9.1 (8.2–10.2)	2.8 (2.3–3.5)	75.6 (73.8–77.2)	12.5 (11.2–13.9)	
High	9.5 (8.1–11.1)	2.1 (1.4–3.1)	77.7 (75.2–80.0)	10.7 (9.0–12.8)	
**Monthly household income^b^**					
					
Low	5.3 (4.3–6.5)	3.0 (2.2–4.1)	74.5 (71.9–76.9)	17.2 (15.1–19.6)	<0.001
Moderate	7.1 (6.1–8.2)	3.6 (2.3–5.5)	77.6 (75.3–79.8)	11.7 (10.1–13.5)	
High	9.3 (8.0–10.9)	2.2 (1.6–3.0)	78.8 (76.7–80.8)	9.6 (8.2–11.2)	
Not reported	7.7 (6.4–9.2)	2.6 (1.7–3.9)	78.1 (75.1–80.8)	11.7 (9.6–14.2)	
**Cigarettes smoked/day**					
<10	11.7 (10.6–13.0)	2.7 (3.4–36.7)	71.6 (69.5–73.5)	14.0 (12.6–15.6)	<0.001
11–20	4.8 (4.1–5.5)	3.1 (2.2–4.2)	82.1 (80.4–83.8)	10.0 (8.7–11.5)	
21–30	2.6 (1.7–4.0)	2.5 (1.5–4.0)	81.6 (77.9–84.8)	13.4 (10.5–16.8)	
31+	3.6 (2.0–6.5)	5.1 (2.6–9.7)	76.5 (70.4–81.7)	14.7 (10.8–19.9)	

a Population size estimate rounded to one decimal place (note: for each individual variable listed the population estimate size may differ due to weights, and these are not reported).

b Data on income were missing from 18.8% (n=2018) of the sample (similar percentage in each tobacco flavour group; including this response option did not impact on significance levels). Income was assessed in each country differently, and a standardized measure was used across countries for comparison: The Netherlands, Ireland, Scotland, and the rest of the UK were asked about their monthly gross household income, while respondents from France and Germany reported their monthly net household income. The response options for the income question were not the same across the countries. Therefore, within each country, a three-level income variable was created: low (<€1750 for Germany, Greece & Spain, ≤150000 Ft for Hungary, ≤2000 zł for Poland, ≤1000 lei for Romania), moderate (€1750 to €3000, 150001 Ft to 250000 Ft, 2001 zł to 4000 zł, 1001 lei to 2500 lei) and high (>€3000, >250000 Ft, >4000 zł, >2500 lei). This approximated an even distribution as closely as possible, which allowed comparisons to be made across countries.

c Rao-Scott chi-squared in complex samples Crosstabs. CI: confidence interval.

**Table 3:** Sociodemographic characteristics of smokers of different cigarette flavours

	*Menthol (n=795.2)^a^*	*Other flavoured (n=311.5)^a^*	*Unflavoured tobacco (n=8298.3)^a^*	*No usual brand (n=1321.5)^a^*	*Total (n=10726.5)^a^*	*p^c^*

	*% (95% CI)*					
**Sex**						
Male	33.0 (29.0–37.2)	59.9 (53.9–65.5)	56.2 (55.0–57.5)	62.6 (59.6–65.4)	55.4 (54.3–56.5)	<0.001
Female	67.0 (62.8–71.0)	40.1 (34.5–46.1)	43.8 (42.5–45.0)	37.4 (34.6–40.4)	44.6 (43.5–45.7)	
**Age (years)**						
18–24	19.4 (16.3–22.9)	14.6 (9.6–21.7)	10.8 (9.9–11.7)	17.4 (14.9–20.1)	12.4 (11.5–13.2)	<0.001
25–39	39.8 (35.6–44.2)	28.3 (20.7–37.2)	30.4 (29.0–31.8)	28.7 (25.5–32.0)	30.8 (29.6–32.0)	
40–54	23.0 (19.8–26.5)	35.8 (29.0–43.3)	31.9 (30.7–33.2)	26.7 (23.8–29.9)	30.7 (29.6–31.8)	
55+	17.8 (15.0–21.1)	21.3 (17.0–26.4)	26.9 (25.7–28.2)	27.3 (24.4–30.3)	26.1 (25.0–27.2)	
**Level of education**						
Low	14.0 (11.7–16.7)	34.9 (22.8–49.3)	31.8 (30.4–33.2)	30.4 (27.2–33.8)	30.4 (29.2–31.6)	<0.001
Moderate	67.8 (64.0–71.5)	54.6 (42.5–66.3)	54.0 (52.5–55.4)	57.0 (53.5–60.5)	55.4 (54.1–56.7)	
High	18.1 (15.5–21.2)	10.5 (7.2–15.0)	14.3 (13.3–15.2)	12.6 (10.5–15.0)	14.2 (13.4–15.1)	
**Monthly household income^b^**						

Low	15.1 (12.3–18.4)	22.0 (16.1–29.3)	20.4 (19.2–21.7)	29.7 (26.3–33.2)	21.2 (20.1–22.4)	<0.001
Moderate	32.1 (28.1–36.3)	41.2 (30.8–52.5)	33.6 (32.1–35.2)	31.8 (282–35.6)	33.5 (32.0–35.0)	
High	33.0 (28.9–37.4)	19.8 (14.3–26.7)	26.7 (25.3–28.1)	20.5 (17.7–23.5)	26.2 (25.0–27.5)	
Not reported	19.8 (16.5–23.4)	17.0 (11.1–25.2)	19.2 (17.8–20.8)	18.1 (14.8–21.9)	19.1 (17.7–20.5)	
**Cigarettes smoked/day**						
<10	64.5 (60.4–68.4)	36.7 (29.6–44.5)	37.3 (35.9–38.7)	46.9 (42.9–51.0)	40.5 (39.2–41.7)	<0.001
11–20	30.7 (27.0–34.6)	49.6 (41.6–57.6)	50.0 (48.6–51.4)	39.1 (35.3–43.0)	47.3 (46.0–48.5)	
21–30	3.0 (2.0–4.6)	7.2 (4.6–11.2)	9.0 (8.3–9.8)	9.5 (7.5–12.0)	8.6 (7.9–9.3)	
31+	1.8 (1.0–3.3)	6.4 (3.4–11.9)	3.6 (3.1–4.2)	4.5 (3.2–6.2)	3.7 (3.2–4.2)	

a Population size estimate rounded to one decimal place (note: for each individual variable listed the weighted population estimate size differed due to weights; these individual estimates are not reported).

b Data on income were missing from 18.8% (n=2018) of the sample (similar percentage in each tobacco flavour group; including this response option did not impact on significance levels). Income was assessed in each country differently, and a standardized measure was used across countries for comparison: The Netherlands, Ireland, Scotland, and the rest of the UK were asked about their monthly gross household income, while respondents from France and Germany reported their monthly net household income. The response options for the income question were not the same across the countries. Therefore, within each country, a three-level income variable was created: low (<€1750 for Germany, Greece & Spain, ≤150000 Ft for Hungary, ≤2000 zł for Poland, ≤1000 lei for Romania), moderate (€1750 to €3000, 150001 Ft to 250000 Ft, 2001 zł to 4000 zł, 1001 lei to 2500 lei) and high (>€3000, >250000 Ft, >4000 zł, >2500 lei). This approximated an even distribution as closely as possible, which allowed comparisons to be made across countries.

c Rao-Scott chi-squared in complex samples Crosstabs. CI: confidence interval.

**Table 4:** Attitudes of smokers towards tobacco control measures (% of smokers supporting each of the measures)

	*Menthol (n=795.2)^c^*	*Other flavoured (n=311.5)^c^*	*Unflavoured tobacco (n=8298.3)^c^*	*No usual brand (n=1321.5)^c^*	*Total (n=10726.5)^c^*	*p^d^*
**Declaring support for:^a^**	% (95% CI)					
**Ban on additives**	25.1 (21.8–28.8)	49.9 (42.2–57.7)	47.9 (46.3–49.6)	48.0 (44.3–51.7)	46.3 (44.8–47.8)	0.000
**Plain packaging**	33.2 (29.3–37.4)	31.4 (24.1–39.7)	28.2 (26.7–29.7)	34.2 (30.6–37.9)	29.4 (28.0–30.8)	0.003
	***Menthol (n=362.1)^c^***	***Other flavoured (n=233.5)^c^***	***Unflavoured tobacco (n=5667.9)^c^***	***No usual brand (n=960.5)^c^***	***Total (n=7224.0)^c^***	***p^d^***
**Smoking ban in restaurants^b^**	73.5 (67.7–78.7)	63.3 (54.1–71.1)	64.7 (62.6–66.7)	68.2 (63.6–72.6)	65.6 (63.7–67.4)	0.044
**Smoking ban in bars and pubs^b^**	56.1 (49.9–62.1)	45.4 (34.9–56.3)	46.4 (44.2–48.6)	52.2 (47.3–57.1)	47.6 (45.6–49.6)	0.021
**More regulation on tobacco^b^**	48.9 (43.0–54.8)	65.7 (53.0–76.4)	45.5 (43.3–47.7)	45.7 (41.0–50.5)	46.4 (44.3–48.4)	0.003

a Table presents percentage of those who reported support for each measure vs all other answers grouped together (i.e. answers indicating no support, ‘don’t know’ and ‘refused’ answers, and missing data).

b Questions not asked in England (n=3536).

c Population size estimate rounded to one decimal place.

d Rao-Scott chi-squared in complex samples Crosstabs.

### Attitudes towards tobacco control measures

Smokers of different cigarette flavours differed significantly in their attitudes towards all tobacco control measures (all p<0.05 from overall chi-squared test, Table 4). All groups were predominantly supportive of a smoking ban in restaurants, with highest support reported by menthol smokers (73.5%), and lowest by other flavoured cigarette smokers (63.3%). Almost half of all smokers supported the ban on smoking in bars and pubs, with the support varying significantly by the type of cigarette smoked (56.1% among menthol, 45.4% among other flavoured cigarette smokers). There was least support for plain packaging, although the support among menthol (33.2%) and other flavoured (31.4%) cigarette smokers was somewhat higher than among unflavoured cigarette smokers (28.2%). Almost half of all smokers agreed that there should be more rules and regulations on tobacco products, with support for this statement particularly high among other flavoured cigarette smokers (65.7%), lower among menthol smokers (48.9%), and lowest among unflavoured cigarette smokers (45.5%). The most divisive issue between menthol smokers and other smokers was that of banning additives, including flavourings, in cigarettes, with only 25.1% of menthol smokers supporting such a law, compared to 49.9% of other flavoured cigarette smokers.

### Intentions regarding smoking behaviour following ban on additives

When asked about their intentions following a ban on the sale of their preferred cigarette brands, menthol smokers and other flavoured cigarette smokers provided different answers, but there was no consistent pattern in their reported intention across countries (Table 5). Overall, 20% of menthol smokers and 23.4% of other flavoured smokers indicated that they would switch to another brand, with the highest percentages observed among Romanian (menthol 45%, other flavoured 33.5%) and German smokers (menthol 40.2%, other flavoured 49.3%). In contrast, only 13.1% of English menthol smokers and 10.1% of Polish other flavoured cigarette smokers indicated that they would switch brands in case of a ban.

**Table 5:** Intentions following bans on menthol and flavoured cigarettes assessed among smokers of menthol^a^ or other flavoured^b^ cigarettes

	*Switch to another brand*	*Find way to get banned product*	*Reduce amount smoked*	*Quit smoking*	*Do something else*	*Don’t know*

	*% (95% CI)*					
**Germany**						
Menthol	40.2 (21.9–61.8)	35.2 (18.6–56.4)	8.5 (2.6–24.0)	2.1 (0.3–12.6)	5.3 (1.3–18.9)	8.7 (2.8–23.8)
Other flavoured	49.3 (31.1–67.7)	11.0 (3.0–33.0)	14.8 (6.3–31.0)	9.5 (3.2–25.1)	7.6 (1.1–38.5)	7.9 (3.1–18.5)
**Greece**						
Menthol	16.8 (5.0–43.5)	20.4 (10.0–37.2)	14.8 (7.0–28.7)	17.3 (5.0–45.2)	6.0 (1.1–26.5)	24.7 (10.0–49.3)
Other flavoured	20.8 (8.3–43.3)	0	14.8 (5.6–33.8)	10.9 (3.9–26.6)	18.1 (6.6–41.1)	35.4 (20.9–53.2)
**Hungary**						
Menthol	21.4 (9.9–40.5)	29.0 (18.2–42.9)	14.1 (6.1–29.4)	16.6 (8.1–31.1)	4.1 (1.2–12.4)	14.7 (6.6–29.8)
Other flavoured	27.3 (12.9–48.8)	15.7 (5.7–36.6)	21.5 (9.0–43.1)	15.8 (5.5–37.7)	6.0 (0.8–33.1)	13.5 (5.5–29.8)
**Poland**						
Menthol	26.4 (17.3–38.2)	12.1 (6.4–21.5)	23.8 (15.4–34.8)	16.0 (9.2–26.3)	3.8 (1.6–8.9)	17.9 (11.4–27.0)
Other flavoured	10.1 (3.4–26.3)	22.3 (11.5–38.9)	21.7 (9.8–41.5)	6.0 (2.2–15.4)	15.4 (4–9–39.1)	24.5 (7.5–56.6)
**Romania**						
Menthol	45.0 (32.9–57.8)	10.8 (4.8–22.5)	15.2 (8.0–26.9)	14.8 (7.6–26.8)	3.4 (1.0–11.1)	10.8 (4.9–22.0)
Other flavoured	33.5 (14.9–59.2)	8.4 (1.1–43.3)	17.2 (6.2–39.7)	17.3 (4.9–46.3)	16.0 (5.1–40.4)	7.5 (1.0–39.1)
**Spain**						
Menthol	0	26.7 (3.8–76.9)	17.5 (2.3–66.0)	11.0 (1.3–52.7)	0	44.9 (11.3–83.9)
Other flavoured	1.1 (0.1–8.7)	0	1.8 (0.2–13.8)	2.3 (0.3–17.1)	86.3 (62.4–96.0)	8.5 (2.1–28.6)
**England**						
Menthol	13.1 (9.9–17.0)	31.7 (26.5–37.4)	18.3 (14.1–23.6)	17.5 (13.8–21.9)	5.2 (2.9–8.9)	14.3 (10.6–19.0)
Other flavoured	28.3 (17.3–42.7)	12.0 (5.7–23.6)	19.2 (10.4–32.5)	15.1 (6.8–30.5)	8.6 (3.8–18.3)	16.8 (8.8–29.7)
**NL**						
Menthol	25.5 (16.2–37.7)	35.3 (24.4–47.9)	12.9 (6.9–22.7)	11.7 (6.3–20.8)	0	14.7 (7.7–26.4)
Other flavoured	26.4 (3.0–80.6)	0	0	0	0	73.6 (19.4–97.0)
**Total**						
Menthol	20.0 (16.9–23.4)	27.3 (23.7–31.3)	17.6 (14.5–21.1)	16.0 (13.3–19.2)	4.3 (2.8–6.5)	14.8 (12.0–18.0)
Other flavoured	23.4 (17.1–31.3)	8.4 (5.2–13.4)	14.7 (10.1–20.9)	10.4 (6.5–16.2)	25.1 (14.2–40.3)	18.0 (12.6–25.0)

a Among all menthol cigarette smokers, 4 refused to answer and were excluded from the analysis.

b Among all other flavoured cigarette smokers, 1 refused to answer and was excluded from the analysis.

Among menthol smokers, 27.3% indicated that in case of a ban they would find a way to get the banned product regardless, compared to 8.4% of other flavoured cigarette smokers. This further varied by country, with a third of German (35.2%), Dutch (35.3%) and English (31.7%) menthol smokers declaring this, compared to only 10.8% Romanian menthol smokers. Among other flavoured cigarette smokers, those from Romania were also least likely to indicate that they would find a way to get the banned product (8.4%), compared with 22.3% of Polish smokers.

A minority of smokers indicated that the menthol and flavoured cigarettes ban would likely lead them to smoke less. Among menthol smokers, 17.6% declared that they would reduce the amount they smoked in case of a ban, and 16% declared that they would quit altogether. Among other flavoured cigarette smokers, the figures were 14.7% and 10.4%, respectively. Intentions to reduce smoking and to quit were most prevalent among menthol smokers in Poland, where 23.8% declared they would reduce smoking, and 16% that they would quit, compared with only 8.5% and 2.1% in Germany, respectively. Among other flavoured cigarette smokers, the highest rate of intention to reduce or quit smoking was observed among Hungarian (21.5% and 15.8%) and English (19.2% and 15.1%) smokers, compared to only 1.8% and 2.3% in Spain.

Throughout the sample, a considerable proportion of smokers declared that in response to the bans they would do something else than the options they were offered (4.3% menthol, 25.1% other flavoured), or that they do not know what they would do (14.8% menthol, 18.0% other flavoured).

## DISCUSSION

The 8E results suggest that the proportion of MFC use in Europe is substantial, but that important differences exist across countries. In addition, smokers of menthol and smokers of other flavoured cigarettes are not a homogenous group, as one might have expected in terms of sociodemographic and nicotine dependence characteristics. With a 7.4% prevalence of menthol use among respondents, and a 2.9% prevalence of other flavoured cigarettes, data suggest that the ban on flavourings introduced by the TPD (postponed to 2020 for menthols) is likely to directly affect at least one in ten smokers throughout the countries surveyed. However, the proportion of menthol use in our study was lower than in the US, where estimates^13^ put menthol cigarette prevalence among past 30-day smokers in 2012–2014 at 39%. England and Poland’s status as countries with the highest rates of menthol use in Europe, with a prevalence of over 10%, corresponds with a Philip Morris report from 2010, as do the marginal rates of menthol use in Spain^17^. The high proportion of menthol smokers in England (15%) found in this study remains particularly noteworthy and might be due to the recent increase in the use of capsule cigarettes; to date flavoured cigarette use has not been investigated by the major cross-sectional surveys in England (e.g. The Smoking Toolkit Study, General Household Survey).

The findings that the use of other flavoured cigarettes in Europe is lower than that of menthol cigarettes explain the industry’s intense lobbying for a postponement of the introduction of the TPD menthol cigarette ban in Europe in 2020. However, in Germany, Greece and Spain, other flavoured cigarette use was actually higher than the rate for menthols, and in the latter two countries, it was above 5%, which confirms the desirability of the bans introduced by the TPD.

It is also useful to compare the prevalence found in this study with previous studies analysing MFC use in individual countries. The results for Romania, with a menthol prevalence of 7.8%, are lower than in a recent study among moderate smokers, which identified a 30% prevalence of menthol use in Romania^30^. The results for Poland, with an MFC use estimated at 6.8% among male and 21.1% among female respondents, are also slightly lower than reported in a study from 2014 using data from the global adult tobacco survey, which put the figures at 10.5% for men and 26.1% for women^31^. This suggests that further studies conducted with larger samples of smokers, or, given the cost and challenges of such studies, research collecting specific samples, for example only menthol smokers, might be needed in order to obtain a more accurate picture of MFC use in individual countries.

The study demonstrates that a sizeable minority of MFC smokers are already well positioned to benefit from the EU TPD. Almost one-third of respondents anticipate that they will reduce their smoking, or quit altogether, in case of a flavouring ban. However, the majority of MFC smokers in the survey still declared little interest in changing their smoking patterns following a ban on flavourings. A survey conducted among young adult (18–34 years old) menthol cigarette smokers in the US found that as many as 66% of respondents would quit smoking if menthol cigarettes were no longer sold^15^. This indicates that there is room for improvement in shaping the quit intentions of European MFC smokers. Without dedicated campaigns promoting cessation among MFC smokers, the TPD ban on flavourings might constitute a missed opportunity for public health.

An important finding of this study is that women have a higher prevalence of MFC use than men (13.7% vs 7.5%), which echoes findings from the US^13^ and Poland^31^. This means that women will be particularly affected by the TPD provisions. This provides an opportunity for tailored cessation support activities targeting female MFC smokers in the wake of the ban. This could be especially important, as the smoking rates among women in Europe have not been declining as rapidly as those among men, and in some countries have not been declining at all^32^.

Amongst the 8E countries where public health could gain most from the ban are England and Poland, where the proportion of smokers self-identifying as MFC smokers is higher than in the other countries. These are therefore the countries where special care should be taken to support cessation efforts in the wake of the TPD ban. While the high levels of MFC use in Poland have been previously documented^17,18,31^, the high levels of menthol use among ITC respondents in England are a novel finding and warrant further research.

A potentially surprising finding was that on some of the measures (e.g. on smoking dependence on attitude on bans) smokers of other flavoured cigarettes were actually more similar to smokers of unflavoured tobacco than to menthol smokers. Further research exploring why this is the case is recommended. Meanwhile, given these differences, care should be taken to avoid grouping together all smokers of flavoured cigarettes in analysis with the assumption that they are a homogenous group.

The study also shows that almost half of all smokers support the ban on additives, including one-quarter of menthol smokers and half of other flavoured cigarette smokers. This is a positive finding, also in comparison with a 2016 US survey that showed a low support of 17% for a ban on menthol cigarettes among US smokers^14^. The majority of MFC smokers tend to be supportive of other tobacco control measures, including smoking bans in restaurants, bars, and pubs, but not on plain packaging, which shows that there still exists a need for better public communication in arguing for certain areas of tobacco control legislation.

However, the fact that 27.3% of menthol smokers declared they would find a way to get the banned product is of concern ahead of the introduction of the menthol ban. In addition, such high prevalence of these attitudes could be used by the TTCs to renew their lobbying against the ban and stoke the fears of negative economic effects that it might have. This has been done in the past, a study commissioned by Philip Morris International in 2013 suggested that removing menthol cigarettes from stores would increase preference of smokers to access brands illicitly by 250% in Poland^33^. Fortunately, this is unlikely, since those who currently smoke flavoured cigarettes—predominantly young, well-educated, urban, female smokers—are much less likely to use illicit cigarettes, the smokers of which are typically older, less educated, rural males^21,31^.

### Strengths and limitations

First, despite the initial large sample size, there was a relatively small sample of flavoured and menthol cigarette smokers that could be used in detailed analysis of intentions and views. This has led in some cases to wide confidence intervals, and therefore interpretation of findings of this study must be treated with caution. In the case of some countries, where the survey was completed in presence of a researcher, social desirability bias might have been present, leading the participants to indicate answers that they assumed the researcher would like to hear. Due to its length, the survey also constituted a big time commitment for respondents, possibly leading to some rash answers, and perhaps contributing to the large percentage of respondents choosing ‘don’t know’ or ‘other’ as their answer. In addition, some of the tobacco control measures the participants were asked about, such as the flavouring ban (apart from the ban on menthol cigarettes), were already being implemented in all countries surveyed during the data collection. Since the restrictions have been formally in place from May 2016, ahead of when the survey took place, the prevalence of flavoured tobacco use among smokers surveyed might be lower than it would have been a few months before the TPD ban. Nonetheless, this seems unlikely. The retailers were allowed to continue selling their stocks of non-compliant items, including flavoured tobacco, until May 2017, thus smokers would still be able to access such products at the time of the survey. Finally, the survey only asks about the smokers’ declared intentions (anticipated reactions to regulation), which might or might not predict actual behaviour once the tobacco regulation comes into action.

Nevertheless, this is the first detailed study of menthol and flavoured cigarettes in Europe, giving us an unprecedented insight into the intentions and beliefs of a substantial group of smokers at a crucial time, as their preferred brands are about to be banned. As noted by other researchers, ‘if even a small percentage of all those who say they would quit tobacco actually did so, following a menthol cigarette ban, numerous lives could potentially be saved’^15^.

## CONCLUSIONS

A minority of smokers across Europe smoke menthol cigarettes, with other flavoured cigarettes being even less prevalent. However, there are important differences in prevalence of use of different cigarette flavours in each country. Additionally, the group of MFC smokers are relatively heterogeneous with the sociodemographic and dependence level (cigarettes smoked per day) of flavoured cigarette smokers resembling more closely that of unflavoured tobacco smokers. Nevertheless, the study shows that the ban on flavourings introduced by the EU TPD (extended to 2020 for menthols) will directly affect one in ten smokers throughout the countries surveyed. It also found that a considerable minority of MFC smokers expect to quit smoking after the ban is introduced. MFC use is particularly high among women, smokers 25–54 years old, and smokers in England and in Poland. These findings emphasise the opportunity for targeting those groups with tailored cessation support activities in the wake of the ban on flavourings, but also suggest that country differences may need to be accounted for in any potential interventions.

## CONFLICTS OF INTEREST

The authors declare that they have no competing interests, financial or otherwise, related to the current work. K. Przewoźniak reports grants and personal fees from the Polska Liga Walki z Rakiem (Polish League Against Cancer), outside the submitted work. C. I. Vardavas reports that he is the Strategic Development Editor of TID and that there are no conflicts of interest with this current work. The rest of the authors have completed and submitted an ICMJE form for disclosure of potential conflicts of interest.

## References

[1] Lewis MJ, Wackowski O (2006). Dealing With an Innovative Industry: A Look at Flavored Cigarettes Promoted by Mainstream Brands. American Journal of Public Health.

[2] Sutton CD, Robinson RG (2004). The marketing of menthol cigarettes in the United States: Populations, messages, and channels. Nicotine & Tobacco Research.

[3] Manning KC, Kelly KJ, Comello ML (2009). Flavoured cigarettes, sensation seeking and adolescents’ perceptions of cigarette brands. Tob Control.

[4] Kuiper NM, Gammon D, Loomis B (2018). Trends in Sales of Flavored and Menthol Tobacco Products in the United States During 2011-2015. Nicotine & Tobacco Research.

[5] Kreslake JM, Wayne GF, Connolly GN (2008). The Menthol Smoker: Tobacco Industry Research on Consumer Sensory Perception of Menthol Cigarettes and its Role in Smoking Behavior. Nicotine & Tobacco Research.

[6] Kaleta D, Polanska K, Bak-Romaniszyn L, Wojtysiak P (2016). Perceived Relative Harm of Selected Cigarettes and Non-Cigarette Tobacco Products—A Study of Young People from a Socio-Economically Disadvantaged Rural Area in Poland. International Journal of Environmental Research and Public Health.

[7] Hersey JC, Ng SW, Nonnemaker J (2006). Are Menthol Cigarettes a Starter Product for Youth?. Nicotine & Tobacco Research.

[8] Villanti AC, Richardson A, Vallone DM, Rath JM (2013). Flavored Tobacco Product Use Among U.S. Young Adults. American Journal of Preventive Medicine.

[9] World Health Organization (2015). Fact sheet on ingredients in tobacco products. World Health Organization, ed.

[10] Feirman SP, Lock D, Cohen JE, Holtgrave DR, Li T (2016). Flavored Tobacco Products in the United States: A Systematic Review Assessing Use and Attitudes. Nicotine Tob Res.

[11] Curtin GM, Sulsky SI, Van Landingham C (2014). Patterns of menthol cigarette use among current smokers, overall and within demographic strata, based on data from four U.S. government surveys. Regulatory Toxicology and Pharmacology.

[12] Okuyemi KS, Ebersole-Robinson M, Nazir N, Ahluwalia JS (2004). African-American menthol and nonmenthol smokers: differences in smoking and cessation experiences. Journal of the National Medical Association.

[13] Villanti AC, Mowery PD, Delnevo CD, Niaura RS, Abrams DB, Giovino GA (2016). Changes in the prevalence and correlates of menthol cigarette use in the USA, 2004–2014. Tobacco Control.

[14] Rose SW, Emery SL, Ennett S, McNaughton Reyes HL, Scott JC, Ribisl KM (2015). Public Support for Family Smoking Prevention and Tobacco Control Act Point-of-Sale Provisions: Results of a National Study. American Journal of Public Health.

[15] Wackowski OA, Manderski MTB, Delnevo CD (2014). Young Adults’ Behavioral Intentions Surrounding a Potential Menthol Cigarette Ban. Nicotine & Tobacco Research.

[16] O’Connor RJ, Bansal-Travers M, Carter LP, Cummings KM (2012). What would menthol smokers do if menthol in cigarettes were banned? Behavioral intentions and simulated demand. Addiction.

[17] Oxford Economics (2012). The influence of the availability of menthol cigarettes on youth smoking prevalence. Commissioned by Philip Morris International.

[18] Giovino GA, Sidney S, Gfroerer J (2004). Epidemiology of menthol cigarette use. Nicotine Tob Res.

[19] WHO Study Group on Tobacco Product Regulation (TobReg) (2016). Advisory note: banning menthol in tobacco products.

[20] Borland R, Yong HH, Siahpush M (2006). Support for and reported compliance with smoke-free restaurants and bars by smokers in four countries: findings from the International Tobacco Control (ITC) Four Country Survey. Tobacco Control.

[21] Joosens L, Ross H, Stokłosa M (2014). EU Policy and Cigarette Smuggling: Assessing the Impacts. Workshop on Cigarette Smuggling.

[22] Vardavas CI, Bécuwe N, Demjén T (2018). Study Protocol of European Regulatory Science on Tobacco (EURESTPLUS): Policy implementation to reduce lung disease. Tobacco Induced Diseases.

[23] ITC Project ITC Four Country Smoking and Vaping Survey Wave 1 (2017). Technical Report. University of Waterloo, Medical University of South Carolina, Cancer Council Victoria, King’s College London.

[24] Fong GT, Thompson ME, Boudreau C (2018). The Conceptual Model and Methods of Wave 1 (2016) of the EUREST-PLUS ITC 6 European Countries Survey. Tobacco Induced Diseases.

[25] Fong GT, Cummings KM, Borland R (2006). The conceptual framework of the International Tobacco Control (ITC) Policy Evaluation Project. Tobacco Control.

[26] International Tobacco Control (ITC) Evaluation Project Surveys.

[27] Nagelhout GE, Willemsen MC, Thompson ME, Fong GT, van den Putte B, de Vries Hein (2010). Is web interviewing a good alternative to telephone interviewing? Findings from the International Tobacco Control (ITC) Netherlands survey. BMC Public Health.

[28] Zethof D, Nagelhout GE, de Rooij M (2016). Attrition analysed in five waves of a longitudinal yearly survey of smokers: findings from the ITC Netherlands survey. Eur J Public Health.

[29] ITC Project ITC Netherlands Survey Wave 1 to 8 (2008-2014). Technical Report.

[30] Trofor L, Gherghesanu R, Chirita R, Trofor A (2014). Outcomes of smoking cessation in moderate nicotine dependent smokers. European Psychiatry.

[31] Kaleta D, Usidame B, Szosland-Fałtyn A, MakowiecDąbrowska T (2014). Use of flavoured cigarettes in Poland: data from the global adult tobacco survey (2009-2010). BMC Public Health.

[32] Pisinger C, Jorgensen T, Toft U (2018). A multifactorial approach to explaining the stagnation in national smoking rates. Dan Med J.

[33] SKIM (2013). The impact of a ban on menthol cigarettes on illicit trade in Poland.

